# Spring irrigation with magnetized water affects soil water-salt distribution, emergence, growth, and photosynthetic characteristics of cotton seedlings in Southern Xinjiang, China

**DOI:** 10.1186/s12870-023-04199-7

**Published:** 2023-04-03

**Authors:** Guo Yi, Wang Quanjiu, Wang Kang, Zhang Jihong, Wei Kai, Liu Yang

**Affiliations:** 1 State Key Laboratory of Eco-Hydraulic in Northwest Arid Region of China, Xi’an University of Technology, Xi’an, 710048 China; 2 School of Water Resource and Hydropower, Xi’an University of Technology, Xi’an , 710048 China

**Keywords:** Spring irrigation; magnetized brackish water; soil water-salt distribution; cotton seedling growth; light response curve

## Abstract

**Background:**

Spring irrigation with freshwater is widely used to reduce soil salinity and increase the soil water content in arid areas. However, this approach requires a huge amount of freshwater, which is problematic given limited freshwater resources. Utilizing brackish water for spring irrigation in combination with magnetized water technology may be a promising alternative strategy.

**Results:**

The objective of this study was to evaluate the effects of four spring irrigation methods (freshwater spring irrigation (FS), magnetized freshwater spring irrigation (MFS), brackish water spring irrigation (BS), and magnetized brackish water spring irrigation (MBS)) on soil water and salt distribution, emergence, growth, and photosynthetic characteristics of cotton seedlings. The results showed that for both freshwater and brackish water, magnetized water irrigation can increase the soil water content for improved desalination effect of irrigation water. Additionally, spring irrigation with magnetized water promoted cotton emergence and seedling growth. Compared with FS treatment, cotton finial emergence rate, emergence index, vigor index, plant height, stem diameter, and leaf area index of MFS treatment increased by 6.25, 7.19, 12.98, 15.60, 8.91, and 20.57%, respectively. Compared with BS treatment, cotton finial emergence rate, emergence index, vigor index, plant height, stem diameter, and leaf area index of MBS treatment increased by 27.78, 39.83, 74.79, 26.40, 14.01, and 57.22%, respectively. Interestingly, we found that spring irrigation with magnetized water can increase the chlorophyll content and net photosynthetic rate of cotton seedlings. The rectangular hyperbolic model (RHM), non-rectangular hyperbolic model (NRHM), exponential model (EM), and modified rectangular hyperbolic model (MRHM) were used to fit and compare the cotton light response curve, and MRHM was determined to be the optimal model to fit the data. This model was used to calculate the photosynthetic parameters of cotton. Compared with FS treatment, the net photosynthetic rate (*P*_*nmax*_), dark respiration rate (*R*_*d*_), light compensation point (*I*_*c*_), light saturation point (*I*_*sat*_), and the range of available light intensity (Δ*I*) of MFS were increased by 5.18, 3.41, 3.18, 2.29 and 2.19%, respectively. Compared with BS treatment, the *P*_*nmax*_, *R*_*d*_*, I*_*c*_*, I*_*sat*_ and Δ*I* of MBS were increased by 26.44, 29.48, 30.05, 5.13, and 2.27%, respectively.

**Conclusion:**

The results show that spring irrigation with magnetized brackish water may be a feasible method to reduce soil salt and increase soil water content when freshwater resources are insufficient.

## Background

Cotton (*Gossypium hirsutum* L.) is one of the most important fiber crops worldwide. As the world's largest cotton consumer and the second largest cotton producer, China plays an important role in meeting the world’s growing fiber demand [1, 2]. Since the 1980s, Xinjiang province has become one of the most important cotton-producing regions of China, producing high yield and fine quality cotton because the heat and sunlight conditions of this region are ideal for cotton growth [3–6]. However, with the rapid development of the cotton planting industry, freshwater shortage has become a key challenge for irrigated agriculture [7–10]. To overcome the problem of scarce water resources, drip irrigation under plastic film mulching (i.e. mulched drip irrigation or MDI) has been widely applied in Xinjiang since the 1990s [11–14]. This technology is considered a highly efficient method because it increases water use efficiency, improves fertilizer use efficiency, regulates soil thermal conditions, reduces salt leaching, and increases cotton production [15–18]. However, some studies have reported that MDI increases the risk of secondary soil salinization due to the limited amount of irrigation water, thus limiting the sustainable development of agriculture [19–21].

In order to solve the problem of secondary salinization of cotton field, salt leaching is widely performed in the non-growth period (spring irrigation or winter irrigation) in local agricultural production practice. Hu et al. (2015) reported that MDI combined with winter or spring irrigation did not result in the significant accumulation of salt in the soil root zone on a multi-year scale [19]. Yang et al. (2016) assessed the effect of different winter irrigation water levels on soil salinity and observed increased desalination effect of winter irrigation with increasing irrigation levels [22]. Both spring irrigation and winter irrigation require a large amount of freshwater [23, 24]. The total annual water consumption of cotton fields in southern Xinjiang is basically maintained at 6000–8250 m^3^·hm^−2^, and 3000–4500 m^3^·hm^−2^ is annually applied by irrigation for salt pressing in cotton fields, accounting for more than 50% of the annual irrigation quota of cotton fields [25]. Given this volume of water, it is clear that non-growth period irrigation aggravates the supply and demand contradiction of limited freshwater resources. The utilization of brackish water is considered an important strategy to alleviate the contradiction between supply and demand of agricultural freshwater resources worldwide [26, 27]. However, brackish water irrigation may aggravate the accumulation of salt in the soil and also cause changes in the chemical composition of the soil solution and the soil physical structure, thus affecting the movement of water and salt in the soil, the soil air permeability, and the extension of plant roots [28–31]. Thus, strategies that utilize brackish water must meet the water demand of crops and control the harm of brackish water.

In recent years, magnetized water technology has been developed to solve the secondary salinization of soil caused by brackish water irrigation [32, 33]. When water passes through a magnetic field, its physical and chemical properties change, which is beneficial to the leaching of soil salt. Mostafazadeh-Fard et al. (2012) demonstrated that magnetized irrigation water used in trickle irrigation has a high potential for reducing soil cations and anions, lowering salt concentrations and improving soil conditions for plant growth [34]. Zlotopolski (2017) reported that magnetized brackish water irrigation changed the salt distribution between soil layers in the soil column, reduced the salt content in the upper layer, and leached more salt into the deep layer of the soil [35]. Moreover, researchers found that magnetized brackish water irrigation can improve the germination characteristics of crop seeds and increase crop yield. For example, Abedinpour and Rohani (2017) reported that the emergence and growth indices of maize were significantly improved by using magnetized saline water [36]. Zhang et al. (2022) reported that, the cotton seed germination rates under magnetized fresh water and magnetized brackish water irrigation increased by 13.14% and 41.86% compared with fresh water and brackish water, respectively [37]. Zhou et al. (2022) showed that compared with unmagnetized brackish water, cotton yield was increased by 31.3% under magnetized brackish water irrigation [33]. The above studies either focus on seed germination or on the stage of growth from seedling to maturity. However, as far as we know, no research has focused on the effects of magnetized water on the sowing to seedling stage. This may be because for most crops, only low amounts of water are required in this stage. However, as mentioned above, spring irrigation before sowing consumes a lot of water for cotton cultivation in Xinjiang. Considering the shortage of fresh water resources in Xinjiang, we wanted to investigate whether the combination of brackish water and magnetized water technology can replace fresh water for spring irrigation.

The objectives of this work were (1) to investigate the effects of magnetized water spring irrigation on the distribution of soil salt and soil water, (2) to evaluate the effects of magnetized water spring irrigation on cotton seedling emergence and seedling growth, (3) to evaluate the effects of magnetized water spring irrigation on the photosynthetic characteristics of cotton seedling.

## Materials and methods

### Experimental site description

The experimental site is located at the key irrigation station (41°35′N, 86°09′E, and 901 m a.s.l.) of the Ministry of Water Resources of People’s Republic of China in Korla City, Xinjiang. After obtaining permission from the Bayingolin Administration Bureau in Tarim River Basin of Xinjiang Uygur Autonomous Region, we carried out field experiments. This area has a typical mainland climate with four distinct seasons, strong solar radiation, and sufficient sunlight. The annual precipitation is approximate 53.5 ~ 62.7 mm and the maximum potential evaporation (20 cm diameter evaporation pan) is 2273 ~ 2788 mm. The maximum, minimum, and annual mean air temperatures are 41.2, -30.5, and 12.5℃, respectively. The average annual number of sunshine hours is 3036.2 h and the frost-free period is 144 ~ 241 d [38]. During the cotton seeding stage, total precipitation was 0.2 mm in 2020. Freshwater and brackish water used for irrigation are from the Peacock River and local groundwater, respectively. The electrical conductivity (EC) and major ion contents of freshwater and brackish water were determined and are presented in Table 1. The soil texture of the area is sandy soil. The soil series is brown-desert soil. Detailed soil physical properties are shown in Table 2.

**Table 1:** Electrical conductivity (EC) and major ion contents of freshwater and brackish water

Water type	EC (µs cm-^1^)	Major anions			Major cations			
		Na^+^ + K^+^	Mg^2+^	Ca^2+^	HCO^3−^	CO^2−^	Cl^−^	SO_4_^2−^
Freshwater	953.25b	89.49b	34.79b	56.17b	205.55b	15.59b	97.6b	145.87b
Brackish water	3027.42a	281.16a	75.94a	118.5a	386.1a	21.12a	249.0a	473.8a

Different letters within a column indicate significant differences among all treatments at *p* < 0.05

**Table 2:** Initial physical properties of soil

Determination	Soil depth (cm)				
	0–20	20–40	40–60	60–80	80–100
Sand (%)	83.1	89.5	88.6	85	82.1
Silt (%)	15.3	9.7	10.5	13.3	16.2
Clay (%)	1.6	0.8	0.9	1.7	1.8
Soil texture	Loamy sand	Sandy	Sandy	Loamy sand	Loamy sand
${\theta }_{S}$(cm^3^/cm^3^)	38.62	34.12	36.99	37.21	36.43
${\theta }_{FC}$(cm^3^/cm^3^)	25.10	17.01	19.81	20.72	21.2
${\theta }_{PWP}$(cm^3^/cm^3^)	3.91	4.26	4.31	4.06	3.8
BD (g/cm3)	1.47	1.64	1.54	1.53	1.55

${\theta }_{S}$, saturated moisture; ${\theta }_{FC}$, field capacity;${\theta }_{PWP}$, permanent wilting point; BD, bulk density

### *Experimental setup*

**Fig. 1:**
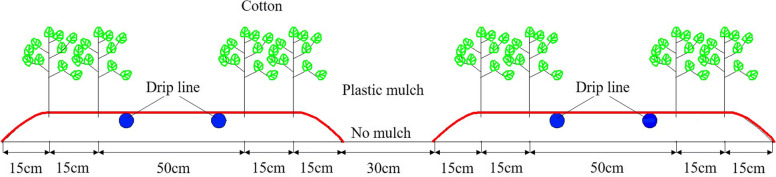
Schematic diagram of cotton planting pattern

The commercial cotton (var. Chuangmian 50) seeds used in this experiment were purchased from Biocentury Transgene (China) Co., Ltd., Xinjiang Uygur Autonomous Region, China. According to the local planting practice, to ensure the normal emergence of cotton seeds, a pre-irrigation step is required before sowing in the spring to leach the soil salt. The pre-irrigation step is defined as spring irrigation. The four spring irrigation treatments applied in this study were: freshwater spring irrigation (FS), magnetized freshwater spring irrigation (MFS), brackish water spring irrigation (BS), and magnetized brackish water spring irrigation (MBS). All treatments were arranged in a randomized block designed with five replicates. Each plot was 10*5.6 m and adjacent plots were separated by 2 m to eliminate the effect of lateral movement of soil water. Spring irrigation was performed with an irrigation amount of on May 6, 2020 [16]. After spring irrigation, inorganic fertilizer (225 kg ha^−1^ urea, containing 46.4% N, 375 kg ha^−1^, diammonium phosphate, 46% P_2_O_5_ and 18% N, and 300 kg ha^−1^ potassium sulfate, containing 45% K_2_O) was applied. The cotton seeds were planted on May 10, 2020 in 'one film, two drip lines and four rows' planting mode. The cotton was drip-irrigated and a submerged pump was used to apply pressure. Four rows of cotton were covered by one white plastic film of 110-cm width and irrigated with two drip lines. Drip lines were 50 cm apart, the distance between emitters is 30 cm, and the flow rate of emitters is 2.2 L/h. Cotton plants were spaced 10 cm apart, with a planting density of 2.2*10^5^ plants/ha. A schematic illustrating the planting pattern and drip line arrangement of cotton at the site is presented in Fig. 1.

**Fig. 2:**
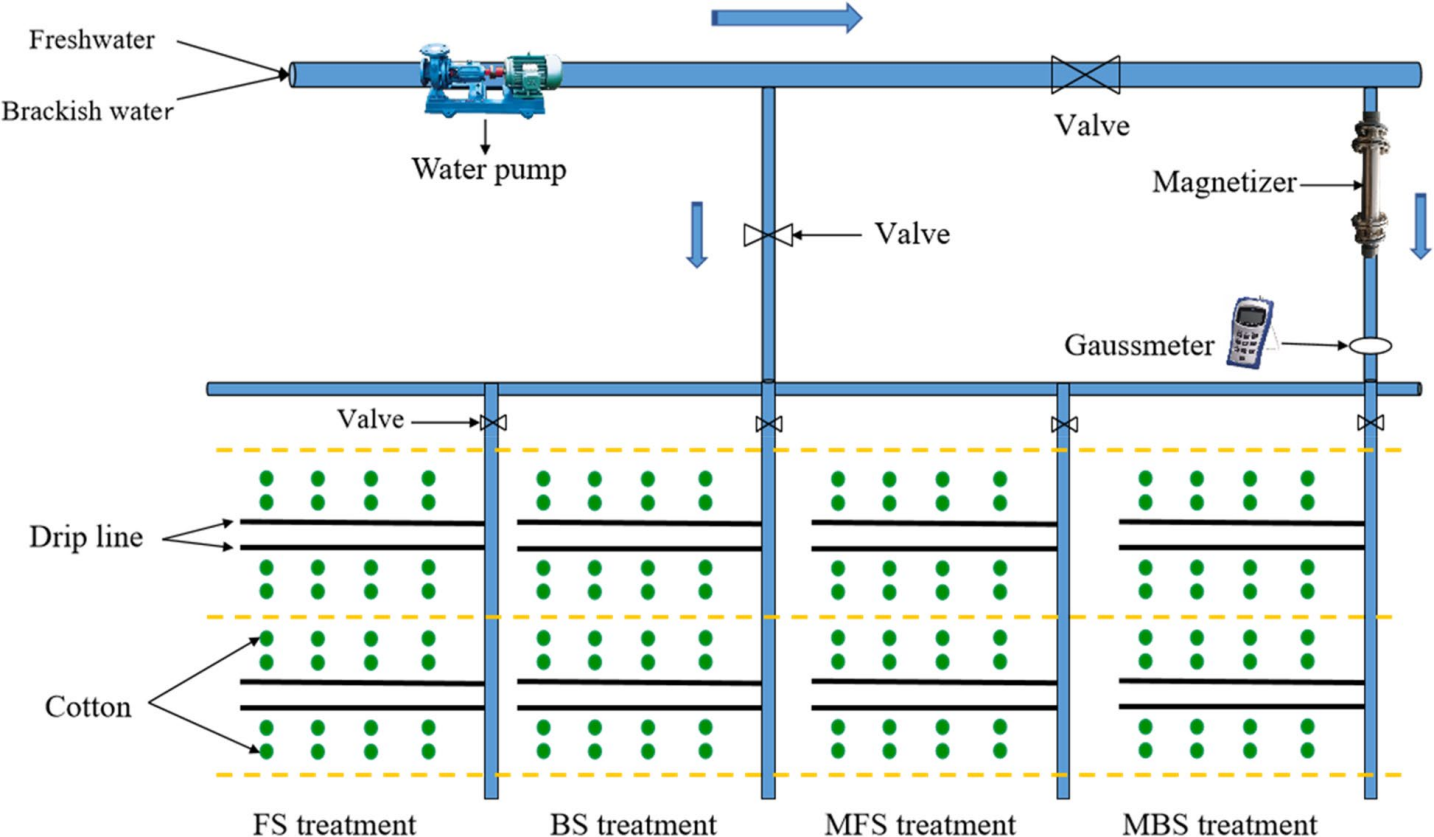
Drip irrigation system arrangement of the experiments

In this design, the spring irrigation water entered each experiment field from pipes equipped with permanent magnets (Baotou Xinda Magnetic Material Factory, China). The effective magnetic field strength of each magnet was calibrated to 3000 GS by a 5180 Gauss/Tesla meter (F. W. Bell Inc., Milwaukee, USA). This field intensity was selected based on previous findings that this magnetic field strength is ideal for cotton growth [32, 39]. Brackish water and freshwater were magnetized during passage through the pipe. The drip irrigation system arrangement is illustrated in Fig. 2.

### Data collection and calculations

#### Soil moisture and soil salinity measurements

Soil water content and salt content were measured before and after spring irrigation (May 6th and 10th) and the cotton seedling stage (June 20th). Soil samples were collected from the cotton root zone with a 5 cm diameter auger to determine the soil moisture and salt content. A 10 cm collection interval was used for the 0 to 40 cm soil samples and a 20 cm collection interval was used for 60–100 cm soil samples. The soil mass water content was determined by the drying method (105 ℃ ± 2℃, 24 h), and the soil volume water content was obtained by multiplying the mass water content and the average bulk density of 0–120 cm soil layer. The dried soil samples were ground and leached at to a soil–water mass ratio of 1:5. After the leaching solution was allowed to stand for eight hours, the electrical conductivity (EC_1:5_) was measured with a DDS-307 conductivity meter (Shanghai Precision & Scientific Instrument Inc., Shanghai, China). Based on a linear relationship (SC = 4.25*EC_1:5_; R^2^ = 0.987; n = 26), the EC_1:5_ value for each soil sample was converted into soil salt content [16].

#### Cotton growth index and physiological index measurement 

The seedling emergence was recorded at intervals of 2 days after sowing, and the seedling emergence rate was calculated. Plant height, stem diameter, maximum leaf length, leaf width, and leaf number were measured every 10–15 days. The plant height, maximum leaf length and leaf width were measured with a tape with an accuracy of 1 mm, and the stem diameter was measured with an electronic vernier caliper with an accuracy of 0.01 mm. The leaf area was calculated as maximum leaf length × width of one side of each leaf multiplied by a factor of 0.703 [38]. Leaf area index (LAI) was calculated by multiplying the total green leaf area per plant by the actual plant density.

The chlorophyll content was determined in SPAD units and measured using SPAD-502 Minolta chlorophyll meter (Spectrum Technologies, Plainfield, USA) at three-leaf, five-leaf, and seven-leaf stages. The light response curve was determined using LCPro-SD portable photosynthesis system (ADC BioScientific, Hoddesdon, UK) on a clear and cloudless morning at the seven-leaf stage of the cotton seedlings. Three leaves in each treatment were randomly selected for measurement. Light was measured in gradient order of 2000, 1800, 1600, 1400, 1200, 1000, 800, 600, 400, 200, 150, 80, 40, and 0 μmol·m^−2^·s^−1^.

### Calculation and analysis methods

#### Salt accumulation calculation

To analyze the effect of magnetized freshwater and magnetized brackish water on salt accumulation at cotton seedling stage, soil salt accumulation was calculated by the salt balance formula as follows:

1 $$\Delta S={S}_{F}-{S}_{I}=\sum_{i=1}^{n}10*{z}_{i}*\overline{\rho }*\Delta {S}_{i}$$

where ${S}_{I}$ is the initial soil salinity, g·m^−2^; ${S}_{F}$ is the final soil salinity, g·m^−2^. ${z}_{i}$ is the thickness of the soil layer, cm; $\overline{\rho }$ is the average bulk density of 120 cm soil section; and $\Delta {S}_{i}$ is the change of soil salt content in the $i$ soil layer. A positive value of $\Delta S$ indicates that the soil is in the state of salt accumulation and a negative $\Delta S$ value indicates that the soil is in a desalination state.

#### Cotton emergence characteristics

The number of emerging cotton seeds was recorded at a intervals of 2 days after sowing. The daily emergence rate, emergence index, and vitality index were calculated as follows:

2 $$DER\left(\%\right)={NSE}_{n}/NST*100$$

3 $$EI=\sum \left({NSE}_{n}/n\right)$$

4 $$VI=EI*SL$$

where $\mathrm{DER}$ is the daily emergence rate (0 ≤ DER ≤ 100%); ${NSE}_{n}$ is the total number of seeds emerging in n days; $NST$ is the number of seeds tested; n is the number of days; $EI$ is the emergence index; $VI$ is the vitality index; and $SL$ is the seedling length.

A logistic model was used to describe the emergence process of cotton seeds [40],

5 $$Y=\frac{K}{1+a{e}^{bt}}$$

where, $K$ is the maximum emergence rate; $t$ is the number of days after sowing, day; $a$ and $b$ are empirical parameters.

Based on formula (5), the following emergence characteristic parameters were calculated as follows:

6 $${t}_{1}=\frac{1}{b}\mathrm{ln}(\frac{2+\sqrt{3}}{a})$$

7 $${t}_{2}=\frac{1}{b}\mathrm{ln}(\frac{2-\sqrt{3}}{a})$$

8 $${t}_{3}={t}_{2}-{t}_{1}$$

9 $${t}_{m}=-\frac{lna}{b}$$

10 $${V}_{m}=-\frac{bK}{4}$$

11 $${V}_{t}=\frac{{y}_{2}-{y}_{1}}{{t}_{2}-{t}_{1}}$$

where ${t}_{1}$ and ${t}_{2}$ are the start and end day of the fast emergence period, in days; ${t}_{3}$ is the duration of the fast emergence period; ${t}_{m}$ is the highest emergence period, day; ${V}_{m}$ is the highest emergence rate; ${y}_{1}$ and ${y}_{2}$ represent the emergence rates at ${t}_{1}$ and ${t}_{2}$, respectively; ${V}_{t}$ is the average emergence rate from ${t}_{1}$ to ${t}_{2}$.

#### Description of photosynthetic response curve model

Four common light response models were used to simulate the light response curve of cotton seedlings, as described in detail below.

- **(1) Rectangular hyperbolic model**

The rectangular hyperbolic model (RHM) was described by [41]:

12 $${P}_{n}=\frac{\alpha I{P}_{nmax}}{\alpha I+{P}_{nmax}}-{R}_{d}$$

The light compensation point was calculated by:

13 $${I}_{c}=\frac{{R}_{d}{P}_{nmax}}{\alpha \left({P}_{nmax}-{R}_{d}\right)}$$

where ${P}_{n}$ is the net photosynthetic rate, μmol·m^−2^·s^−1^; $\alpha$ is the apparent quantum efficiency, which indicates the utilization efficiency of plants in photosynthesis, mol·mol^−^; $I$ is the photosynthetic active radiation (PAR), μmol·m^−2^·s^−1^; ${P}_{nmax}$ is the maximum net photosynthetic rate, μmol·m^−2^·s^−1^; ${R}_{d}$ is the dark respiration rate, μmol·m^−2^·s^−1^.; ${I}_{c}$ is the light compensation point, μmol·m^−2^·s^−1^.

- **(2) Non-rectangular hyperbolic model**

The non-rectangular hyperbolic model (NRHM) was given by [42]:

14 $${P}_{n}=\frac{\alpha I+{P}_{nmax}-\sqrt{{\left(\alpha I+{P}_{nmax}\right)}^{2}-4I\alpha k{P}_{nmax}}}{2k}-{R}_{d}$$

The light compensation point was calculated by:

15 $${I}_{c}=\frac{{R}_{d}{P}_{nmax}-k{R}_{d}}{\alpha \left({P}_{nmax}-{R}_{d}\right)}$$

where *k* is the curved angle of non-right hyperbolic curve, $0<k\le 1$.

- **(3) Exponential model**

The exponential model (EM) was given by [43]:

16 $${P}_{n}={P}_{nmax}\left(1-Q{e}^{\frac{-\alpha I}{{P}_{nmax}}}\right)$$

where *Q* is a constant that represents the net photosynthetic rate approaching zero under weak light.

The light compensation point was calculated by:

17 $${I}_{c}=\frac{{P}_{nmax}\mathrm{ln}(Q)}{\alpha }$$

- **(4) Modified rectangular hyperbolic model**

The modified rectangular hyperbolic model (MRHM) was calculated by [44]:

18 $${P}_{n}=\alpha \frac{1-\beta I}{1+\gamma I}I-{R}_{d}$$

where *β* is a correction factor, *γ* is a coefficient that is independent of $I$.

${P}_{nmax}$, ${I}_{c}$ and ${I}_{sat}$ were calculated by:

19 $${P}_{nmax}=\alpha {\left(\frac{\sqrt{\beta +\gamma }-\sqrt{\beta }}{\gamma }\right)}^{2}-{R}_{d}$$

20 $${I}_{c}=\frac{\alpha -\gamma {R}_{d}-\sqrt{{\left(\gamma {R}_{d}-\alpha \right)}^{2}-4\alpha \beta {R}_{d}}}{2\alpha \beta }$$

21 $${I}_{sat}=\frac{\sqrt{\frac{\beta +\gamma }{\beta }-1}}{\gamma }$$

### Statistical analysis

The statistical analyses were performed using SPSS 22.0 software (IBM Crop., New York, USA). The soil salt amount, emergence rate, emergence index, vitality index, plant height, stem diameter, leaf area index, and SPAD value were measured, and the average values and standard deviations were determined for each treatment (n = 5). Differences were determined using Duncan’s multiple range test at the 5% level of significance. OriginPro 2019 (Origin Lab Corporation, Northampton, MA, USA) was used to graph the data. For the parameters in the plant light response model, OriginPro 2019 was used for curve fitting, and the Levenberg–Marquardt iteration method was used to solve the model parameters.

The initial value and limit range of RHM model parameters were set as follows:

$$\mathrm{Initial value}: \alpha =0.02\mathrm{ mol} {\mathrm{mol}}^{-1},{P}_{nmax}=20\mathrm{ \mu mol}\cdot {\mathrm{m}}^{-2}\cdot {\mathrm{s}}^{-1},{R}_{d}=2 \mathrm{\mu mol}\cdot {\mathrm{m}}^{-2}\cdot {\mathrm{s}}^{-1}$$

$$\mathrm{Limit range}: \alpha \le 1 mol {mol}^{-1},{P}_{nmax}\le 50 \mu mol\cdot {m}^{-2}\cdot {s}^{-1},{R}_{d}\le 12 \mu mol\cdot {m}^{-2}\cdot {s}^{-1}$$

The initial value and limit range of NRHM model parameters were set as follows:

$$\mathrm{Initial value}: \alpha =0.02\mathrm{ mol} {\mathrm{mol}}^{-1},{P}_{nmax}=20\mathrm{ \mu mol}\cdot {\mathrm{m}}^{-2}\cdot {\mathrm{s}}^{-1},{R}_{d}=2 \mathrm{\mu mol}\cdot {\mathrm{m}}^{-2}\cdot {\mathrm{s}}^{-1},k=0.5$$

$$\mathrm{Limit range}: \alpha \le 1 mol {mol}^{-1},{P}_{nmax}\le 50 \mu mol\cdot {m}^{-2}\cdot {s}^{-1},{R}_{d}\le 12 \mu mol\cdot {m}^{-2}\cdot {s}^{-1}; k\le 1$$

The initial value and limit range of EM model parameters were set as follows:

$$\mathrm{Initial value}: \alpha =0.02\mathrm{ mol} {\mathrm{mol}}^{-1},{P}_{nmax}=20\mathrm{ \mu mol}\cdot {\mathrm{m}}^{-2}\cdot {\mathrm{s}}^{-1},Q=1$$

$$\mathrm{Limit range}: \alpha \le 1 mol {mol}^{-1},{P}_{nmax}\le 50 \mu mol\cdot {m}^{-2}\cdot {s}^{-1},Q\le 2$$

The initial value and limit range of MRHM model parameters were set as follows:

$$\mathrm{Initial value}:\alpha =0.02\mathrm{ mol} {\mathrm{mol}}^{-1},\beta =0.0001,\gamma =0.001, {R}_{d}=2 \mathrm{\mu mol}\cdot {\mathrm{m}}^{-2}\cdot {\mathrm{s}}^{-1}$$

$$\mathrm{Limit range}:\alpha \le 1 mol {mol}^{-1},\beta \le 1,\gamma \le 1{;R}_{d}\le 12 \mu mol\cdot {m}^{-2}\cdot {s}^{-1}$$

The determination coefficient (R^2^), the root mean square error (RMSE) and the mean absolute error (MAE) were used to evaluate the accuracy of the light response model. These statistical indexes were calculated as follows:

22 $${R}^{2}=1-\frac{{\sum }_{i=1}^{n}{\left({M}_{i}-{S}_{i}\right)}^{2}}{{\sum }_{i=1}^{n}{\left({M}_{i}-\overline{M }\right)}^{2}}$$

23 $$RMSE=\sqrt{\frac{\sum_{i=1}^{n}{\left({M}_{i}-{S}_{i}\right)}^{2}}{n}}$$

24 $$MAE=\frac{1}{n}{\sum }_{i=1}^{n}\left|{M}_{i}-{S}_{i}\right|$$

where ${M}_{i}$ is the measured value, ${S}_{i}$ is the simulated value, $\overline{M }$ is the average value of the measured data, and n is the number of measurements.

## Results

### *Soil moisture distribution*

**Fig. 3:**
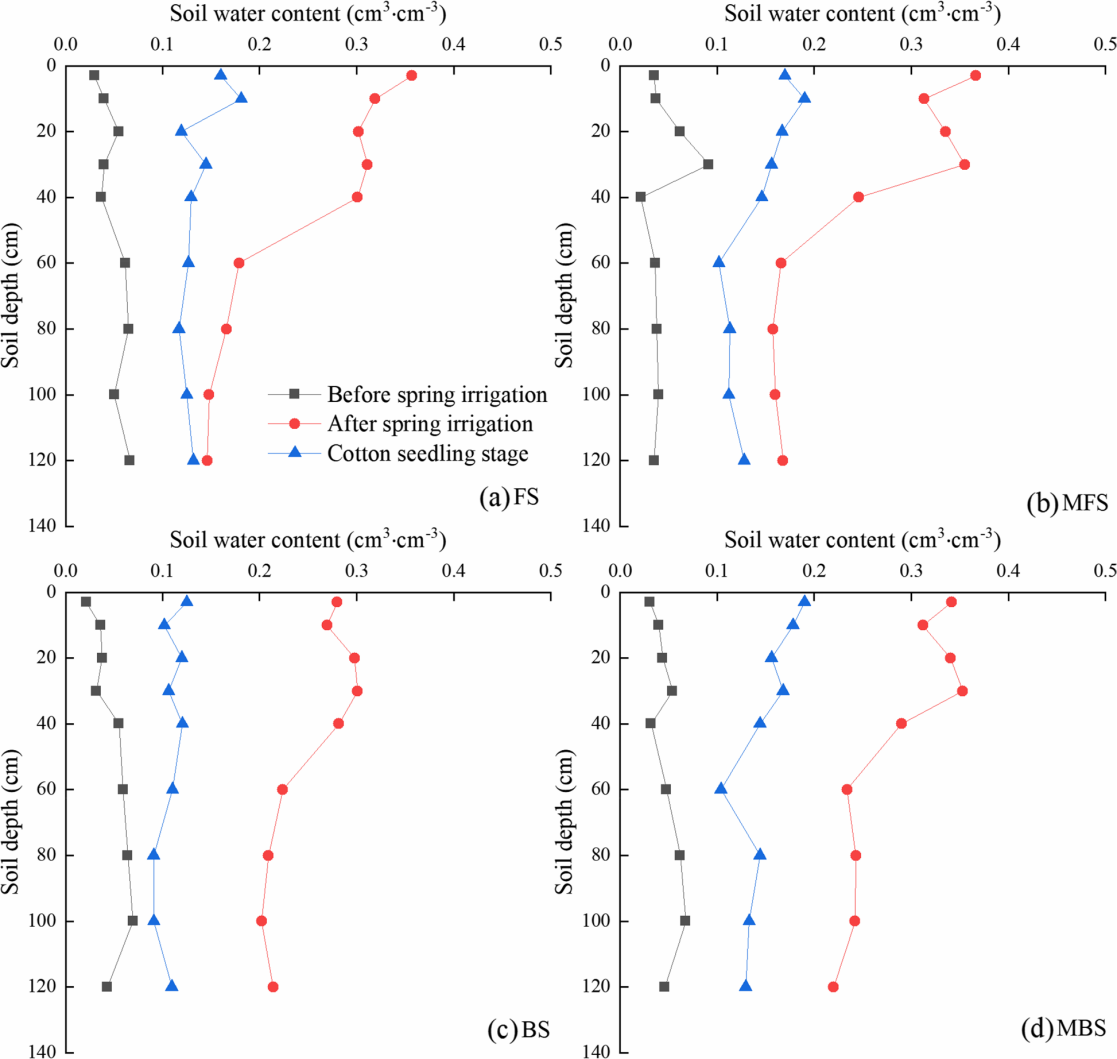
Effect of different spring irrigation treatments on soil moisture distribution. FS is freshwater spring irrigation, MFS is magnetized freshwater spring irrigation, BS is brackish water spring irrigation, and MBS is magnetized brackish water spring irrigation

The vertical distribution of soil water content in the 0–120 cm profile was determined before and after spring irrigation for different treatments, as shown in Fig. 3. The soil moisture content before spring irrigation was 4%-5%, which is close to the wilting water content. After spring irrigation, the average volume water content of 0 ~ 120 cm was about 25%, and the water content of the profile decreased from ground-level and stabilized at 60 cm. At the cotton seedling stage, the average volume water content of 0 ~ 120 cm soil was reduced to 11% ~ 15% due to evaporation at the soil surface. The spring irrigation treatment mainly affects the early growth of cotton, so the soil water content distribution of 0 ~ 40 cm is an important factor affecting early growth. Therefore, we mainly focus on analyzing the distribution of soil water content in the 0–40 cm soil layer. After spring irrigation, the average volumes of freshwater, magnetized freshwater, brackish water, magnetized brackish water treatment were 31.8%, 32.3%, 28.6%, and 32.7%, respectively. At the cotton seedling stage, the average volumes of freshwater, magnetized freshwater, brackish water, and magnetized brackish water were 14.7%, 16.6%, 11.5%, and 16.7% in the 0–40 cm soil layer, respectively. The water content was greater for the magnetized freshwater and magnetized brackish water treatments than for the unmagnetized freshwater and unmagnetized brackish water treatments within 0 ~ 40 cm.

### Soil salt distribution and accumulation

**Fig. 4:**
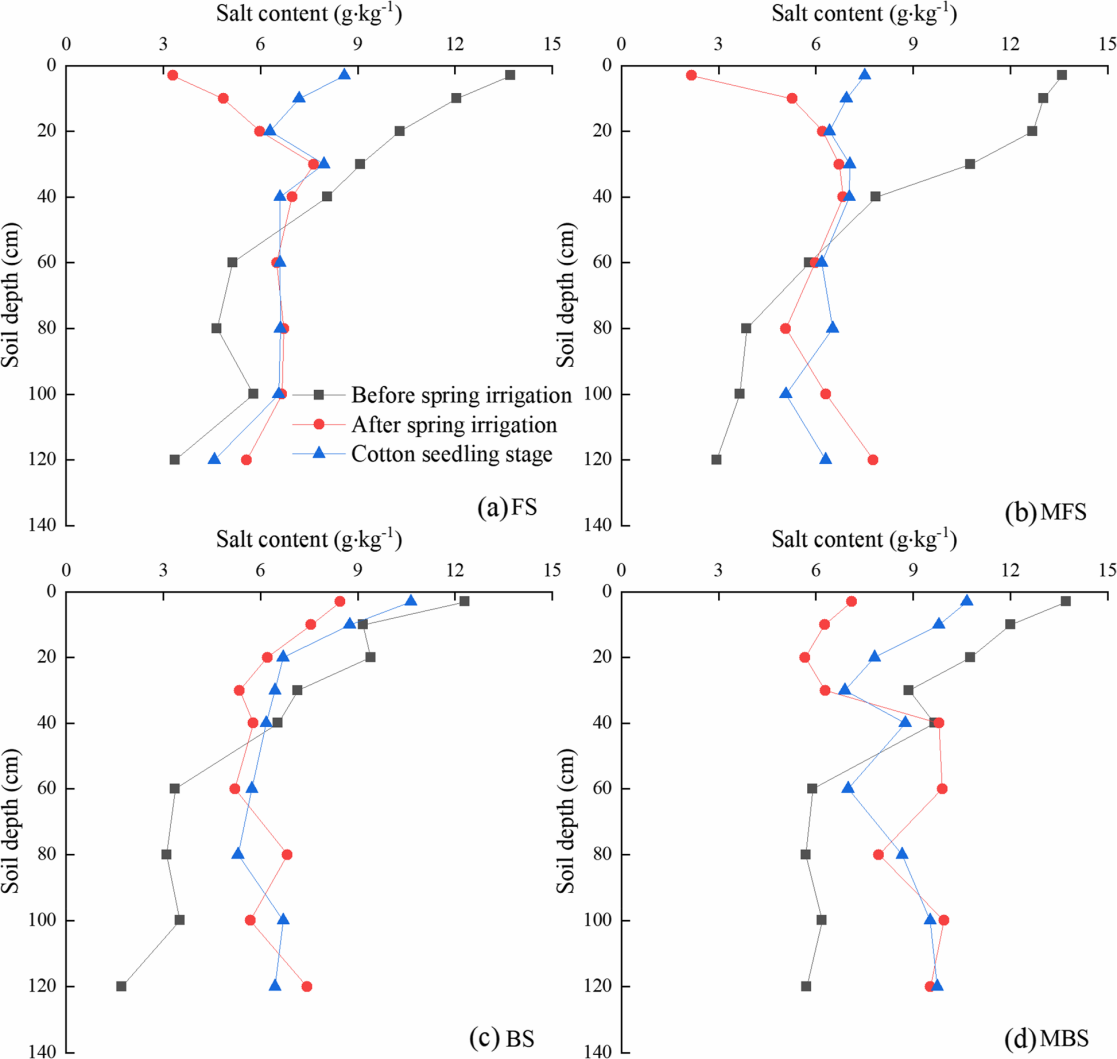
Effect of different spring irrigation treatments on soil salt distribution. FS is freshwater spring irrigation, MFS is magnetized freshwater spring irrigation, BS is brackish water spring irrigation, and MBS is magnetized brackish water spring irrigation

The vertical distribution of soil salt of the 0–120 cm profile was measured before and after spring irrigation for different treatments and the results are shown in Fig. 4. The growth of cotton seedlings occurs mainly in the 0–40 cm soil layer, so we analyzed the salt distribution in this soil layer. The average salt content of the 0–40 cm soil profile before spring irrigation was 8.22–11.11 g·kg^−1^. After spring irrigation, shallow soil salt was leached into the deep soil, and the average soil salt content of the 0–40 cm soil profile decreased to 6.10–7.05 g·kg^−1^. At the seedling stage, the soil salt content increased to 6.89–8.36 g·kg^−1^ due to increased evaporation. The initial soil salinity content was significantly different for different experimental plots, so the soil salinity accumulation was analyzed to better evaluate the effect of different spring irrigation measures (Table 3). Spring irrigation treatment significantly affected soil salt amount accumulation (*p* < 0.05). Soil salt was leached to the 40–120 cm soil layer under different spring irrigation treatments, with desalination of the 0–40 cm soil layer. We compared the effect of different spring irrigation treatments on the desalination rate of the 0–40 cm soil layer. The treatment effects on desalination rate were in the order of MFS > FS > MBS > BS. After spring irrigation, the soil desalination rate under MFS treatment was 22.19% higher than that of the FS treatment, and the soil desalination rate under MBS treatment was 36.12% higher than that of the BS treatment. At the cotton seedling stage, the soil desalination rate under MFS treatment was 31.36% higher than that of the FS treatment and the desalination rate under MBS treatment was 46.32% higher than that of the BS treatment.

**Table 3:** Soil salt balance for all treatments in 0–40 cm, 40–120 cm, and 0–120 cm soil profiles

Soil profile	Treatme nt	*S*_*i*_ (g·m^−2^)	$\Delta$ *S*_*1*_ (g·m^−2^)	Accumulation rate	$\Delta$ *S*_*2*_ (g·m^−2^)	Accumulation rate
0–40 cm	FS	6811.19 b	-2512.86 c	-36.89 c	-1971.06 c	-28.94 c
	MFS	7601.85 a	-3426.79 d	-45.08 d	-2889.78 d	-38.01 d
	BS	5623.89 c	-1335.61 a	-23.74 a	-760.55 a	-13.52 a
	MBS	7124.97 ab	-2303.27 b	-32.33 b	-1409.85 b	-19.79 b
40–120 cm	FS	6482.52 b	2221.65 c	34.28 d	1842.01 d	28.42 c
	MFS	5564.29 c	3036.43 b	54.57 c	2671.16 c	47.99 b
	BS	4002.51 d	4598.48 a	114.87 a	4272.42 a	106.73 a
	MBS	8042.82 a	4726.03 a	58.77 b	3904.86 b	48.56 b
0–120 cm	FS	13,293.72 b	-291.21 c	-2.19 c	-129.05 c	-0.97 c
	MFS	13,166.14 b	-390.37 c	-2.96 d	-218.62 d	-1.67 d
	BS	9626.4 c	3262.87 a	33.89 a	3511.87 a	36.48 a
	MBS	15,167.77 a	2422.76 b	15.97 b	2495.01 b	16.45 b

Different letters within a column indicate significant differences among all treatments at *p* < 0.05. *S*_*i*_ is the initial soil salt content before spring irrigation, g·m^−2^; $\Delta$ *S*_*1*_ is the change of soil salinity before and after spring irrigation, g·m^−2^; $\Delta$ *S*_*2*_ is the change of salt content at the seedling stage and the initial salt content, g·m^−2^

#### Emergence and cotton seedling growth characteristics

**Fig. 5:**
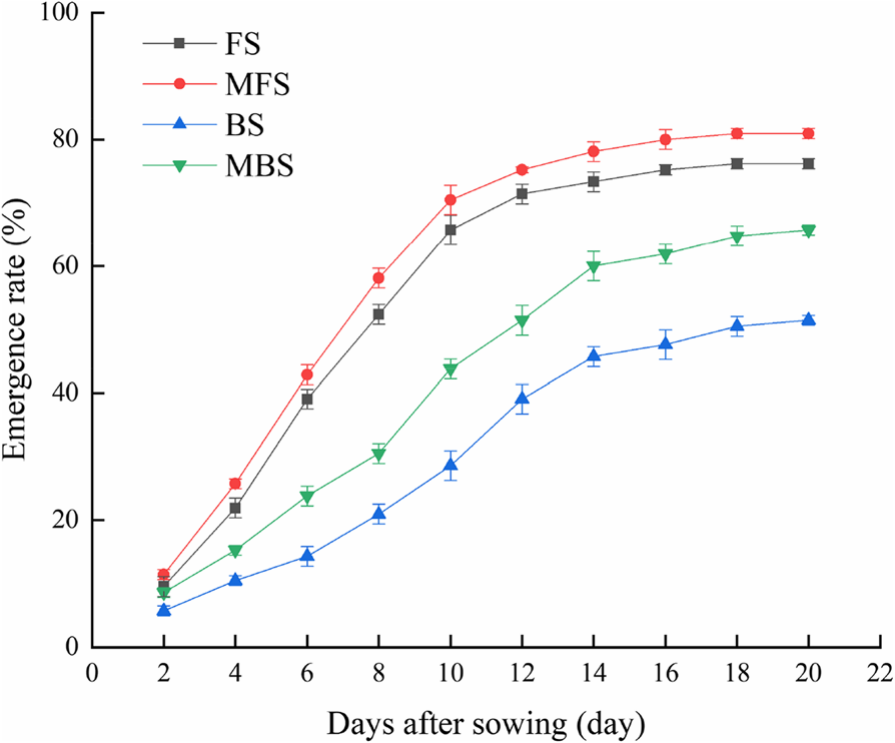
Effect of different spring irrigation treatments on cotton seedling emergence rate. FS is freshwater spring irrigation, MFS is magnetized freshwater spring irrigation, BS is brackish water spring irrigation, and MBS is magnetized brackish water spring irrigation

**Fig. 6:**
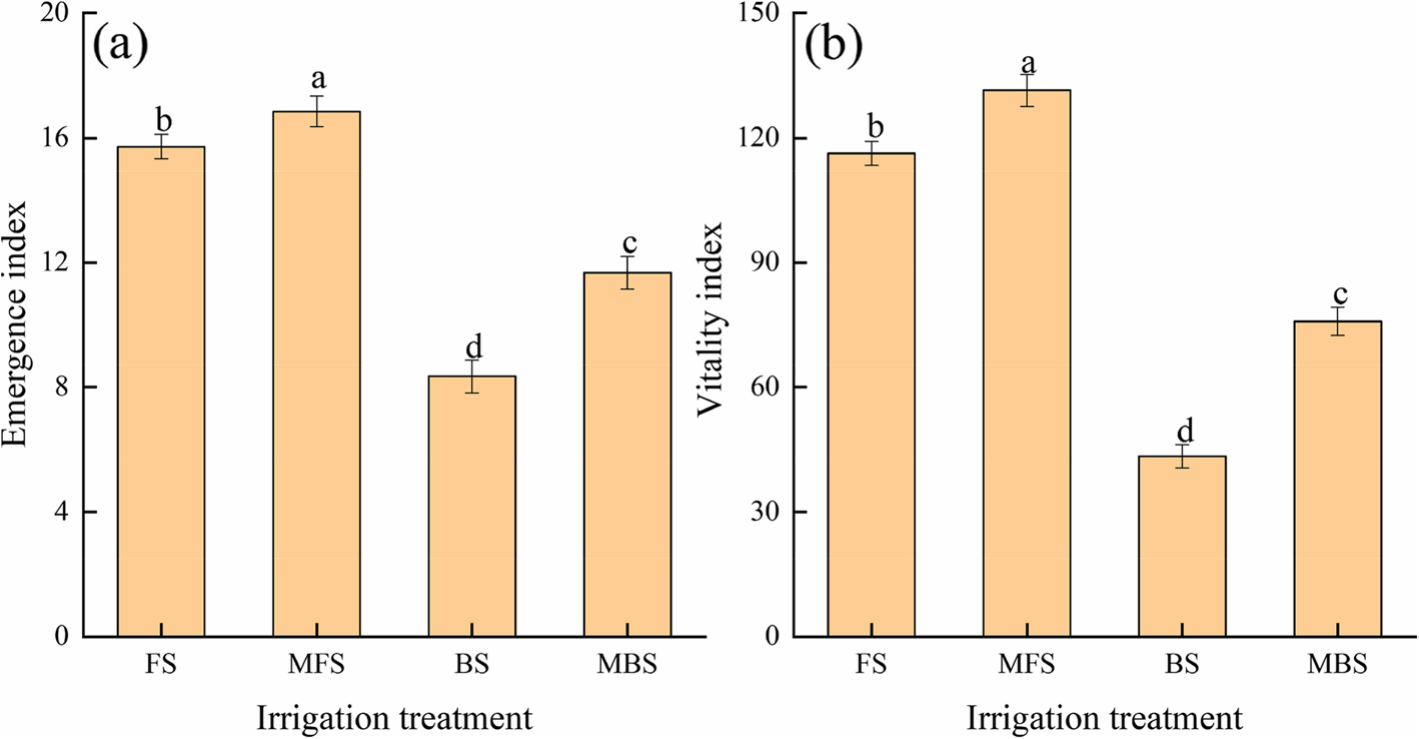
Effect of different spring irrigation treatments on emergence index and vitality index. FS is freshwater spring irrigation, MFS is magnetized freshwater spring irrigation, BS is brackish water spring irrigation, and MBS is magnetized brackish water spring irrigation. Data are mean value of the five replicates. Error bars mean standard errors. Differences were determined using Duncan’s multiple range test. Different letters above the bars indicate significant differences among treatments at *p* < 0.05

The emergence rate of cotton differed among the spring irrigation treatments (Fig. 5). Irrigation water types had significant effects on cotton emergence rate (*p* < 0.05). With increased time, the emergence rate of cotton gradually increased and finally stabilized. The emergence rate of cotton seeds irrigated with brackish water was lower than that of seeds treated with fresh water, and the emergence rate of cotton seeds irrigated with magnetized water was higher than that of seeds treated with non-magnetized water. The order of final emergence rates was MFS > FS > MBS > BS. Compared with fresh water irrigation, the final emergence rate of cotton seeds decreased by 32.5% under brackish water irrigation. Compared with FS and BS treatments, cotton emergence rates increased by 6.25% and 27.78% for MFS and MBS treatments, respectively. The order of both emergence index and vitality index was MFS > FS > MBS > BS (Fig. 6). The emergence index and vitality index of cotton decreased by 46.89% and 62.68%, respectively, under brackish water irrigation compared with fresh water irrigation. Compared with FS, cotton emergence index and vigor index increased by 7.19% and 39.83% under MFS, respectively. Compared with BS, cotton seedling index and vigor index increased by 12.98% and 74.79% under MBS, respectively. The results show that using either brackish water or fresh water for irrigation, magnetic treatment can promote the emergence of cotton seeds.

**Fig. 7:**
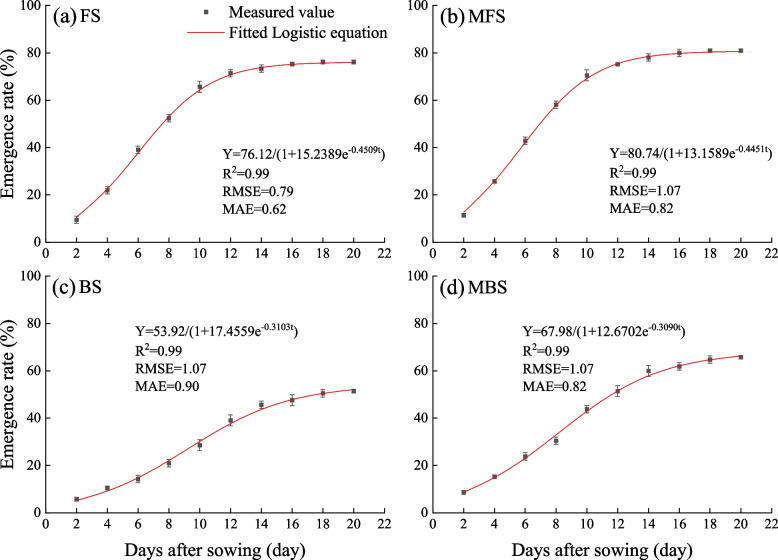
Fitting curve of cotton seed emergence rate with Logistic equation. FS is freshwater spring irrigation, MFS is magnetized freshwater spring irrigation, BS is brackish water spring irrigation, and MBS is magnetized brackish water spring irrigation. Data are mean value of the five replicates. Error bars mean standard errors

Formula (5) was used to quantify the emergence process of cotton seeds. The logistic regression equations under different treatments are shown in Fig. 7. The values of R^2^, RMSE, and MAE indicate that the logistic equation provides a good fit for cotton emergence. The six characteristic parameters associated with emergence are listed in Table 4. Compared with FS, the fast emergence period (${t}_{3}$) of MFS was prolonged by 2.40%, the average emergence rate (${V}_{t}$) of the fast emergence period increased by 4.84%, the highest emergence period (${t}_{m}$) decreased by 4.14%, and the maximum emergence rate (${V}_{m}$) increased by 4.70%. Compared with BS, the fast emergence period (${t}_{3}$) of MBS was prolonged by 2.67%, the average emergence rate (${V}_{t}$) of the fast emergence period increased by 25.90%, the highest emergence period (${t}_{m}$) decreased by 10.83%, and the maximum emergence rate (${V}_{m}$) increased by 25.55%.

**Table 4:** Characteristic parameters of cotton emergence under different irrigation treatments

Treatment	Fast emergence period				Fastest emergence point	
	${t}_{1}$(day)	${t}_{2}$(day)	${t}_{3}$(day)	${V}_{t}$	${t}_{m}$(day)	${V}_{m}$
FS	5.8399	8.9609	3.1210	7.5914	6.0403	8.5813
MFS	5.5532	8.7491	3.1959	7.9588	5.7901	8.9844
BS	8.9643	13.4619	4.4977	3.6958	9.2171	4.1823
MBS	7.8633	12.4811	4.6178	4.6532	8.2185	5.2509

${t}_{1}$ and ${t}_{2}$ are the start and end day of the fast emergence period, day; ${t}_{3}$ is duration of the fast emergence period; ${t}_{m}$ is the highest emergence period, day; ${V}_{t}$ is the average emergence rate from ${t}_{1}$ to ${t}_{2}$; ${V}_{m}$ is the highest emergence rate

**Fig. 8:**
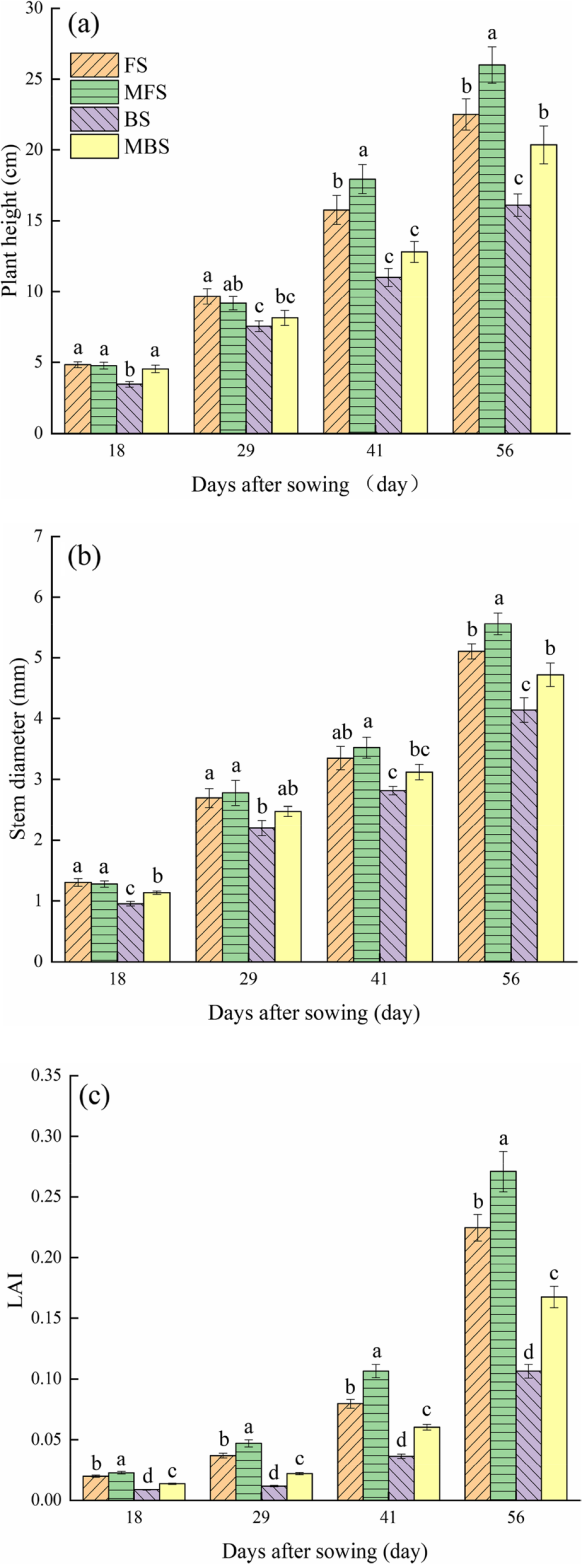
Effect of different spring irrigation treatments on cotton plant height, stem diameter, and leaf area index. Figures 8a, 8b, and 8c are the dynamic trends of plant height, stem diameter and leaf area index with time, respectively. FS is freshwater spring irrigation, MFS is magnetized freshwater spring irrigation, BS is brackish water spring irrigation, and MBS is magnetized brackish water spring irrigation. Data are mean value of the five replicates. Error bars mean standard errors. Differences were determined using Duncan’s multiple range test. Different letters above the bars indicate significant differences among treatments at *p* < 0.05

Figure 8 shows the effects of spring irrigation water type on the growth indexes of cotton seedling. The plant height, stem diameter, and leaf area index of cotton seedlings under brackish water irrigation were significantly lower than those of fresh water (*p* < 0.05). Compared with fresh water irrigation, the plant height, stem diameter, and leaf area index of cotton seedlings under brackish water irrigation decreased by 28.44%, 18.90%, and 52.62% respectively after 56 days of sowing. In addition, magnetization treatment significantly improved the growth indexes of cotton seedlings 56 days after sowing (*p* < 0.05). Compared with freshwater irrigation, plant height, stem diameter, and leaf area index increased by 15.60%, 8.91%, and 20.57%, respectively, after magnetized freshwater irrigation. Compared with brackish water irrigation, plant height, stem diameter, and leaf area index increased by 26.40%, 14.01%, and 57.22%, respectively after magnetized brackish water irrigation.

#### Chlorophyll content and photosynthetic characteristics

**Fig. 9:**
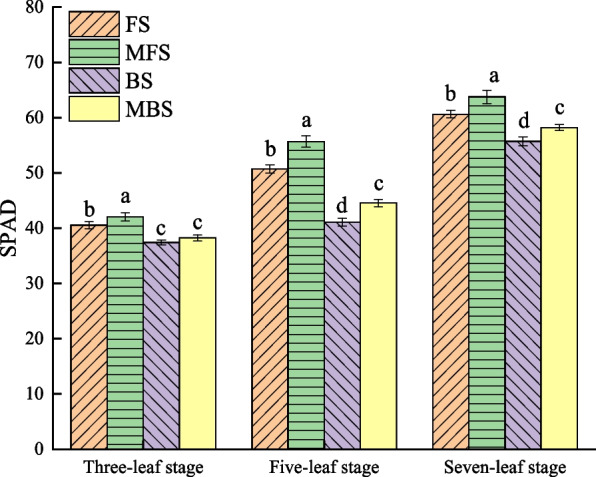
Effect of different spring irrigation treatments on SPAD. FS is freshwater spring irrigation, MFS is magnetized freshwater spring irrigation, BS is brackish water spring irrigation, and MBS is magnetized brackish water spring irrigation. Data are mean value of the five replicates. Error bars mean standard errors. Differences were determined using Duncan’s multiple range test. Different letters above the bars indicate significant differences among treatments at *p* < 0.05

Similar changes in SPAD values of cotton all treatments during the cotton seedling stage (Fig. 9). With the growth of cotton, the SPAD values of cotton seedlings continuously increased. At the seven-leaf stage, the SPAD value under brackish water irrigation was significantly lower than that under fresh water irrigation, and the SPAD value under magnetized water irrigation was significantly higher than that under non-magnetized water irrigation (*p* < 0.05). Thus, the SPAD values of the treatments at the seven-leaf stage were as follows: MFS > FS > MBS > BS. Compared with fresh water irrigation, the SPAD value decreased by 7.12% under brackish water irrigation. Compared with FS and BS, the cotton SPAD values of MFS and MBS treatments increased by 4.58% and 7.13%, respectively.

**Fig. 10:**
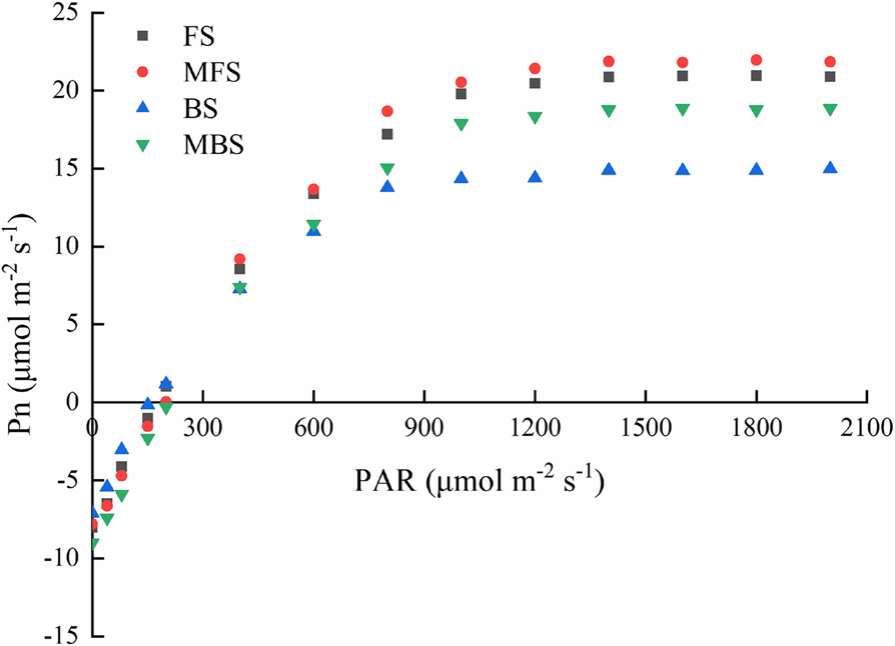
Photosynthesis response process of leaves under different spring irrigation treatments. FS is freshwater spring irrigation, MFS is magnetized freshwater spring irrigation, BS is brackish water spring irrigation, and MBS is magnetized brackish water spring irrigation

The light response process of cotton seedlings was measured under the four spring irrigation treatments, as shown in Fig. 10. Under weak light conditions (PAR ≤ 200 μmol·m^−2^·s^−1^), *P*_*n*_ increased rapidly and linearly with the increase of PAR; under moderate light intensity(200 < PAR ≤ 1000 μmol·m^−2^·s^−1^), the growth rate of *P*_*n*_ decreased; under strong light stage (PAR > 1000 μmol·m^−2^·s^−1^), *P*_*n*_ slowly increased to the light saturation point, eventually reaching the maximum photosynthetic rate. When the PAR exceeded 1600 μmol·m^−2^·s^−1^, there was little change in *P*_*n*_. The *P*_*n*_ was lower under brackish water irrigation than that under fresh water irrigation, and the *P*_*n*_ was higher under magnetized water irrigation than that under non-magnetized water irrigation. The order of *P*_*n*_ was as follows: MFS > FS > MBS > BS.

**Fig. 11:**
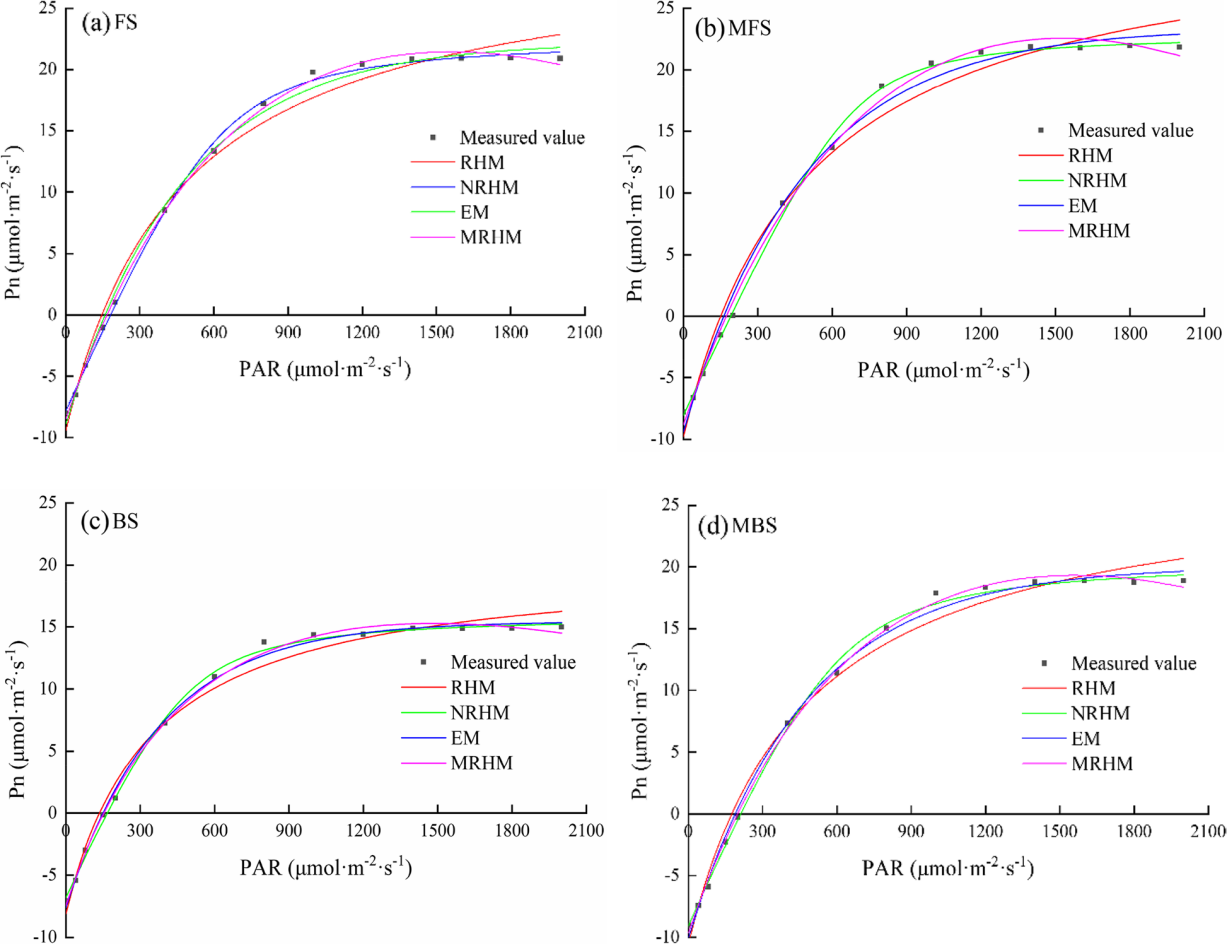
Fitting comparison of four models for light response process of cotton seedlings. FS is freshwater spring irrigation, MFS is magnetized freshwater spring irrigation, BS is brackish water spring irrigation, and MBS is magnetized brackish water spring irrigation. RHM is the rectangular hyperbolic model, NRHM is the non-rectangular hyperbolic model, EM is the exponential model, and MRHM is the modified rectangular hyperbolic model

The light response curve model can be used to determine indicators of the photosynthetic capacity of plants, including apparent quantum efficiency (*α*), dark respiration rate (*R*_*d*_), maximum net photosynthetic rate (*P*_*nmax*_), light compensation point (*I*_*c*_), and light saturation point (*I*_*sat*_). However, it is important to select this best model to fit the data, as different models are based on different mechanisms. Here, we fit the light response data to four common light response models, MRHM, NRHM, EM, and RHM, to compare the photosynthetic physiology of cotton under different spring irrigation treatments (Fig. 11). For PAR ≤ 200 μmol·m^−2^·s^−1^, each model well fit the measured data, but when the PAR > 200 μmol·m^−2^·s^−1^, the simulated curves of each model were significantly different. Table 5 shows the goodness of fit for the four models and the estimation of light response characteristic parameters. Fitting the light response measured data of FS and MBS treatments, the fitting accuracy of the four models was in the order of: MRHM > NRHM > EM > RHM. Fitting the light response measured data of MFS and BS treatments, the fitting accuracy of the four models was in the order: NRHM > MRHM > EM > RHM. However, relying on R^2^, RMSE, and MAE cannot fully assess the quality of model fitting, and other parameters are needed to judge the accuracy of model fitting results.

**Table 5:** Parameter estimation and goodness of fit of different light response curve models for cotton seedlings

Treatment	Empirical model	P_nmax_	R_d_	I_c_	I_sat_	α	R^2^	RMSE	MAE
		μmol·m^−2^·s^−1^							
FS	RHM	39.92	9.47	146.19		0.085	0.988	1.20	1.03
	NRHM	30.47	7.85	181.64		0.045	0.999	0.38	0.33
	EM	22.23	8.96	159.33		0.047	0.997	0.64	0.54
	MRHM	21.44	8.51	167.29	1554.90	0.059	0.999	0.35	0.30
	Measured value	20.89	8.06	174.75	≈1600	0.046			
MFS	RHM	42.24	9.77	149.97		0.085	0.984	1.50	1.26
	NRHM	31.07	8.04	190.61		0.043	0.999	0.43	0.33
	EM	23.41	9.37	162.35		0.049	0.993	0.95	0.78
	MRHM	22.55	8.80	172.61	1590.55	0.058	0.997	0.65	0.55
	Measured value	21.83	8.19	192.18	≈1600	0.043			
BS	RHM	28.50	8.11	135.49		0.084	0.988	0.93	0.77
	NRHM	22.81	6.80	167.24		0.042	0.998	0.34	0.30
	EM	15.43	7.59	149.85		0.041	0.997	0.44	0.34
	MRHM	15.28	7.43	152.77	1482.82	0.060	0.998	0.39	0.32
	Measured value	14.98	7.42	161.27	≈1500	0.046			
MBS	RHM	38.48	10.52	175.18		0.082	0.988	1.17	0.99
	NRHM	29.71	9.05	211.86		0.045	0.998	0.43	0.36
	EM	20.06	10.03	189.12		0.043	0.996	0.64	0.54
	MRHM	19.32	9.62	198.67	1558.94	0.058	0.999	0.41	0.36
	Measured value	18.88	9.15	205.71	≈1500	0.045			

*P*_*nmax*_ reflects a plant’s maximum assimilation capacity [45]. As shown in Table 5, magnetization treatment significantly improved the *P*_*nmax*_ value of cotton seedlings, and the order of fitting results for *P*_*nmax*_ was MRHM > EM > NRHM > RHM. $\alpha$ reflects the ability of plants to absorb, convert, and utilize light energy under low light intensity [46]. The data show a very small effect of magnetization treatment on $\alpha$, almost negligible. The order of fitting results for $\alpha$ was NRHM > MRHM > EM > RHM (Table 5). *R*_*d*_ is the respiration rate of plants in the absence of light, and the dark respiration rate is related to the physiological activity of leaves [47]. Magnetization treatment enhanced *R*_*d*_, indicating that magnetization treatment enhances the physiological activity of leaves. The order of fitting results for *R*_*d*_ from largest to smallest was: NRHM > MRHM > EM > RHM. The *I*_*c*_ and *I*_*sat*_ values reflect the ability of plants to utilize weak and strong light, respectively [46]. Here we define Δ*I* (Δ*I* = *I*_*sat*_- *I*_*c*_) as the range of available light intensity of cotton. The increase of *I*_*c*_ under magnetization treatment indicated that magnetization reduces the ability of plants to utilize weak light. The order of fitting results for *I*_*c*_ from large to small was: NRHM > MRHM > EM > RHM. Since the equations of RHM, NRHM, and EM have no extreme values, the light saturation point cannot be directly calculated. Only MRHM can be used to directly calculate the *I*_*sat*_. The increased values of *I*_*sat*_ and Δ*I* under magnetized water treatment indicated that magnetized irrigation enhanced the ability of cotton plants to utilize strong light and increased the range of cotton's available light intensity. In general, among the four models, MRHM and NRHM exhibited better simulation accuracy for photosynthetic parameters. At the same time, since NRHM fitted *P*_*nmax*_ far beyond the measured value and cannot be used to calculate *I*_*sat*_ (Table 5), MRHM was selected as the most suitable model. Compared with FS, the *P*_*nmax*_, *R*_*d*_, *I*_*c*_, *I*_*sat*_ and Δ*I* values of MFS were increased by 5.18%, 3.41%, 3.18%, 2.29%, and 2.19%, respectively. Compared with BS, the *P*_*nmax*_, *R*_*d*_, *I*_*c*_, *I*_*sat*_, and Δ*I* of MBS were increased by 26.44%, 29.48%, 30.05%, 5.13%, and 2.27%, respectively.

## Discussion

### Effect of the four spring irrigation treatments on soil moisture and salt distribution

In the agricultural production of cotton in Xinjiang, spring irrigation is widely adopted to reduce soil salinity and increase soil water content [22, 48]. In this study, we found higher average water content of 0–40 cm soil under magnetized irrigation than that under non-magnetized irrigation for both freshwater and brackish water. There may be two explanations for the observed increased soil water content after magnetized water irrigation. First, under the action of magnetic field, the average distance between water molecules increases, and some hydrogen bonds become weak or even break, decreasing the size of large associative water molecule clusters with decomposition into free monomer and dimer molecules. These free water molecules can easily enter and be stored in the micro spaces of soil particles. Second, magnetization treatment reduces the water surface tension and viscosity coefficient, decreasing the water infiltration rate and soil hydraulic conductivity, allowing water to remain in the upper layer of the soil, reducing deep leakage, and hindering the evaporation process. Similarly, Mostafazadeh-Fard et al. (2011) reported that the soil water content of 0-60 cm under magnetized water drip irrigation conditions increased by 7.5% compared to the use of non-magnetized water drip irrigation [49]. Zlotopolski (2017) showed that compared with non-magnetized infiltration, magnetized infiltration increased soil water-holding capacity by 25% [35]. Surendran et al. (2016) also found higher soil moisture content after magnetized water drip irrigation compared to the use of non-magnetized water drip irrigation [50]. Zhou et al. (2022) showed that magnetized brackish water irrigation can reduce deep infiltration and hinder evaporation, so more water is retained in the soil profile [33].

In our study, the four spring irrigation treatments significantly reduced the soil salt content with varying effects for the different irrigation methods. Soil salt content in the 0 ~ 40 cm soil was in the order of BS > MBS > FS > MFS, suggesting that magnetized freshwater irrigation and magnetized brackish water effectively increase salt washing efficiency. The smaller molecular cluster and contact angle of magnetized water makes it easier for water to invade the small pores of soil, so that water and soil salt can be combined more effectively, carry more soil salt to migrate with water, and increase the convection and diffusion of soil salt migration for greater washing efficiency. Consistently with our results, Zlotopolski (2017) reported that enhanced leaching of soil salts and ions by magnetized infiltration compared to non-magnetized infiltration [35]. Mostafazadeh-Fard et al. (2011) also found that magnetized water can effectively reduce the SO_4_^2−^ ion content in soil and accelerate salt leaching [49]. Similarly, Zhou et al. (2021) reported that magnetized irrigation water can increase salinity leaching in cotton fields and reduce the stress of salinity on cotton growth [39].

### *Effects of magnetized water irrigation on emergence and seedling growth characteristics*

Cotton emergence and seedling growth are more sensitive to growth environment than later stages of cotton growth [51–53]. For saline alkali soil in arid areas, soil water and salt content are key factors affecting cotton emergence [24]. In this study, the soil salt content before spring irrigation was 0.82–1.11%, and the soil salt content decreased to 0.61%-0.71% after spring irrigation. Some studies have shown that a soil salt content greater than 0.3% affects the emergence of cotton seedlings and a salt content greater than 0.7% severely inhibits germination [54, 55]. Our spring irrigation treatments effectively reduced the soil salt content to the range suitable for cotton seedling emergence. We found that the order of cotton finial emergence rate, emergence index, and vitality index of the four spring irrigation treatments was MFS > FS > MBS > BS. There is clearly an inverse correlation with soil salt content, as the soil salt content after spring irrigation was the highest in BS, second highest in MBS, third highest in FS, and the lowest in MFS. Additionally, the soil water content was highest under MFS treatment and lowest under BS treatment, further explaining the trend for cotton emergence rate. Previous studies have also shown that high soil moisture is beneficial to the emergence of cotton seeds [56–58]. In addition, changes in the physicochemical properties of irrigation water due to magnetization will also affect the emergence of seeds. Zhang et al. (2022) conducted a water absorption experiment on cotton seeds under the conditions of magnetized water and non-magnetized water [37]. The research showed that magnetized water is more easily absorbed by cotton seeds than non-magnetized water. After magnetization, the amount of free monomer and dimer water molecules increases, the surface tension and contact angle decrease, and the osmotic pressure and decompression increase, allowing water to more easily pass through the cell membrane for better water absorption of cotton seeds to promote seed germination and emergence. Consistent with our finding, Zhou et al. (2021) also observed increased emergence rate of cotton with the use of magnetized water for irrigation of saline alkali soil at different levels [39].

Plant height, stem diameter, and leaf area index are important cotton agronomic indicators. In this study, the growth indexes of cotton under fresh water irrigation were better than those under brackish water irrigation. This is because compared with fresh water irrigation, brackish water irrigation does not allow sufficient salt leaching in soil, and the remaining salt in the soil can cause salt stress on cotton seedlings and inhibit the growth of cotton. This is consistent with previous research results [59, 60]. We also found improved cotton growth indicators (plant height, stem diameter and leaf area index) for both magnetized freshwater and magnetized brackish water spring irrigation compared to unmagnetized freshwater and brackish water spring irrigation. Water is abundant in plant cells and is essential for plant metabolism [61]. The magnetization of water changes water’s physical and chemical characteristics (such as surface tension, viscosity coefficient, dissolved oxygen amount and PH value), which can affect crop growth [32]. Magnetized irrigation water promotes the water absorption and utilization of the crops due to lower surface tension and viscosity of magnetized water, and this improves the absorption of nutrients and promotes plant growth [62]. Additionally, the magnetized water exhibits a better salt washing effect and slows the stress of salt on crop growth [63, 64].

### Effects of magnetized water irrigation on chlorophyll content and photosynthetic performance

Crops absorb light energy mainly through chloroplasts, which directly participate in the absorption, transfer, distribution and transformation of light energy in photosynthesis, and the level of chlorophyll content can reflect plant photosynthetic ability [65, 66]. In this study, the SPAD value of cotton was lower under brackish water irrigation than that under fresh water irrigation because salt stress limits the synthesis of chlorophyll in cotton leaves. The reduction in SPAD value under salt stress has been commonly reported [67, 68]. The SPAD value of cotton under magnetized water irrigation at the seven-leaf stage was significantly higher than that under non-magnetized water irrigation. This effect may be because magnetized water increases the activation rates of enzymes and hormones to further promote the production of chlorophyll [32]. In this respect, the results are in agreement with other results obtained in cotton [37, 39], wheat [32], pepper [69], soybean [70], and cowpeas [71].

Photosynthesis is an important physiological process that is sensitive to soil water and salt conditions [72]. In this study, the photosynthesis performance under each treatment ranked as MFS > FS > MBS > BS. Compared with freshwater spring irrigation, brackish water spring irrigation inhibited the photosynthetic performance of cotton. This makes sense because salt stress damages the photosynthetic organs of crops, inhibits or damages the electron transport system of photosynthesis, and reduces the photosynthetic intensity of plants [73, 74]. Our results showed that the magnetization treatment improved the photosynthesis performance of the cotton seedlings compared with unmagnetized treatment. Magnetized water irrigation may enhance the photosynthetic performance of cotton seedlings by increasing the H^+^ proton pump activity of the plants and increasing the free water in the cells to improve the photochemical activity of chloroplasts [75]. Similarly, Liu et al. (2020) reported that magnetization treatment promoted efficient electron transfer between the photosystems of grape seedlings and improved the photosynthetic carbon assimilation ability under salinity stress [76]. Sadeghipour and Aghaei (2013) found that compared with unmagnetized water irrigation, magnetized water irrigation increased the stomatal conductance and photosynthetic rate of cowpea leaves [71]. Anand et al. (2012) also reported an increase in stomatal conductance, photosynthetic rate, and chlorophyll content in maize under magnetized water irrigation [77].

The plant light response curve describes the change of the net photosynthetic rate with the intensity of the light, reflecting the photosynthetic ability of the plant [78–80]. The light response curve model can be used to obtain multiple indicators that characterize the photosynthetic capacity of plants, including apparent quantum efficiency (*α*), dark respiration rate (*R*_*d*_), maximum net photosynthetic rate (*P*_*nmax*_), light compensation point (*I*_*c*_), and light saturation point (*I*_*sat*_). However, different models are based on different mechanisms, so calculation gives different parameter indicators. In this work, four common light response models were used to compare the photosynthetic physiology of cotton under different spring irrigation treatments. The results showed that MRHM was the most suitable model for these data. We found that magnetized water spring irrigation had little effect on *α*, and significantly increased *R*_*d*_, *P*_*nmax*_ and *I*_*sat*_, and Δ*I*, indicating that magnetized water irrigation increased the tolerance of cotton seedlings to strong light and expanded the range of available light intensity. Ning et al. (2020) also reported that magnetized water irrigation could increase the available light intensity range of buckwheat under salt stress [81].

### Correlation analysis

**Fig. 12:**
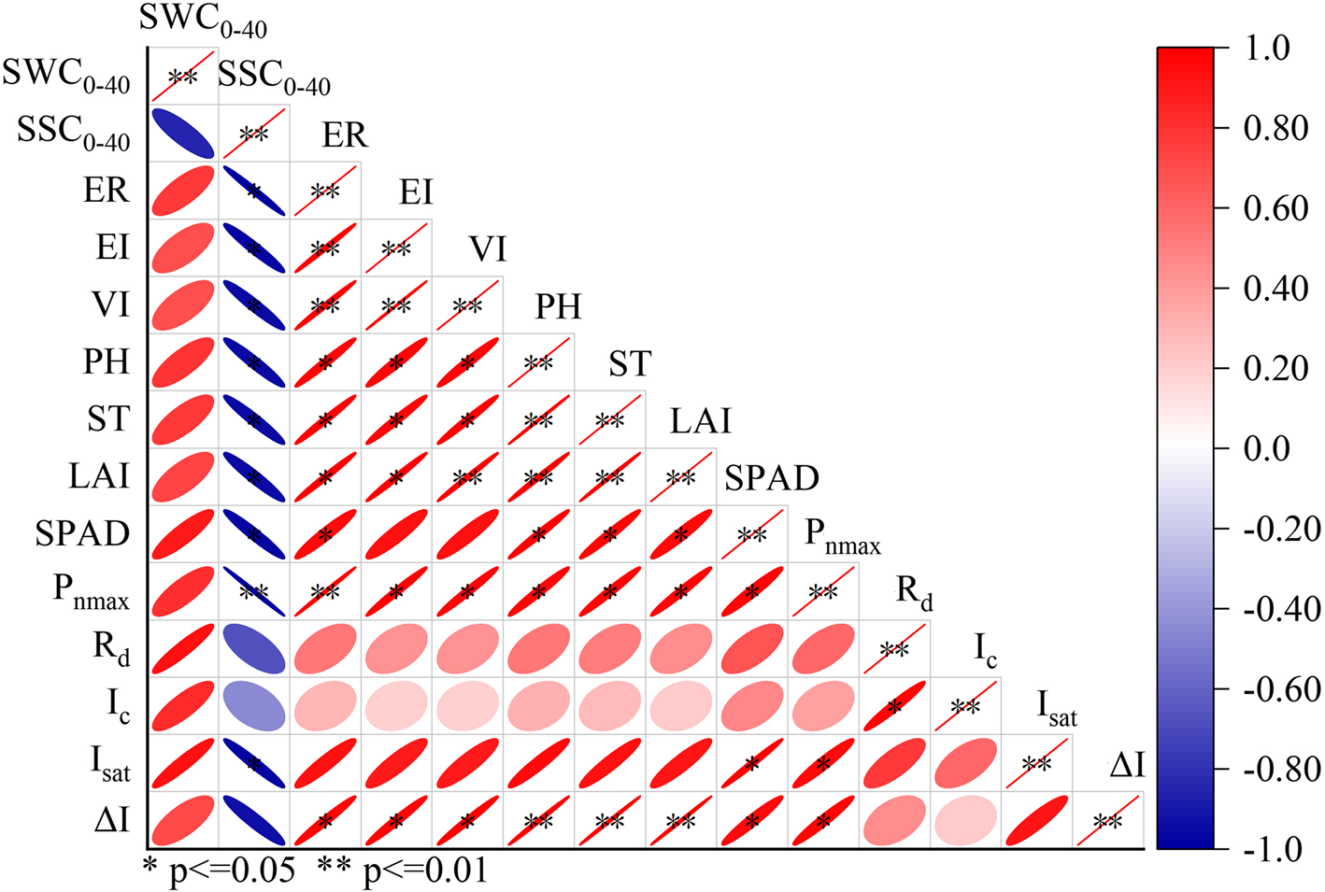
Correlation plot between cotton growth indexes and water and salt content of 0-40 cm soil. * and ** mean significance level of 1% and 5%, respectively. The meaning of the abbreviations: SWC_0-40_ (average soil water content of 0–40 cm soil at the seedling stage), SSC_0-40_ (average soil salt content of 0–40 cm soil at the seedling stage), ER (emergence rate), EI (emergence index), VI (vitality index), PH (plant height), ST (stem diameter), LAI (leaf area index), *P*_*nmax*_ (maximum net photosynthetic rate),* R*_*d*_ (dark respiration rate), *I*_*c*_ (light compensation point), *I*_*sat*_ (light saturation point), Δ*I* (the range of available light intensity). The gradient of the legend is a function of the strength of the correlation, while the slope of the ellipse indicates a negative or positive correlation (i.e., towards the right is a positive correlation, and towards the left is negative correlation). The shape of the ellipse indicates the strength of the correlation

Figure 12 shows the correlation analysis between cotton growth indicators and water and salt content of 0-40 cm soil at the seedling stage. SWC_0-40_ was found to have a positive connection with ER, EI, VI, PH, ST, LAI, SPAD, *P*_*nmax*_*, R*_*d*_*, I*_*c*_*, I*_*sat*_, and Δ*I* (*P* > 0.05). At the same time, SSC_0-40_ was found to have a significant negative connection with ER, EI, VI, PH, ST, LAI, SPAD, and *I*_*sat*_ (*p* < 0.05). In addition, the correlation between SSC0-40 and *P*_*nmax*_ reached a very significant level (*p* < 0.01).

## Conclusion

A field experiment was carried out to study the effects of four spring irrigation methods on soil water and salt environment, cotton emergence characteristics, seedling growth indexes (plant height, stem diameter, and leaf area index) and seedling physiological indicators (SPAD and photosynthetic properties) in southern Xinjiang, China. The results showed that magnetic treatment of irrigation water can increase soil water content, improve the desalting effect of irrigation water, promote cotton seedling emergence and seedling growth, and increase SPAD value and net photosynthetic rate for both brackish water or fresh water irrigation. Especially for brackish water, magnetization treatment greatly improved the emergence characteristics, growth indicators, and physiological indicators of cotton. Among the four common light response models, the MRHM best simulated the light response data under different irrigation treatments. The ranges of R^2^, RMSE, and MAE, respectively, were 0.997–0.999, 0.35–0.65, and 0.30–0.55. The photosynthetic characteristic parameters of cotton calculated based on MRHM showed that magnetization treatment enhanced *P*_*nmax*_, *R*_*d*_, *I*_*c*_, and *I*_*sat*_ compared with unmagnetized treatment. Overall, our work indicates the use of magnetized brackish water spring irrigation may be a feasible strategy to remove soil salt and increase soil water content when freshwater resources are insufficient.

## Data Availability

The data that support the results are included within the article. Other relevant materials are available from the corresponding authors on reasonable request.
